# ROBOT-ASSISTED TARGETED GAIT TRAINING

**DOI:** 10.5604/01.3001.0053.9679

**Published:** 2023-10-26

**Authors:** Vaughn Chambers, Madison Johnson, Panagiotis Artemiadis

**Affiliations:** 1 Mechanical Engineering Department at the University of Delaware, USA

**Keywords:** Human gait, robotics, training, rehabilitation, compliant, stiffness perturbations

## Abstract

**Background:**

Millions of people are affected yearly by “runner’s knee” and osteoarthritis, which is thought to be related to impact force. Millions are also affected by chronic falling, who are usually both difficult to identify and train. While at first glance, these topics seem to be entirely disconnected, there appears to be a need for a device that would address both issues. This paper proposes and investigates the use of the Variable Stiffness Treadmill (VST) as a targeted training device for the different populations described above.

**Materials and Methods:**

The VST is the authors’ unique robotic split-belt treadmill that can reduce the vertical ground stiffness of the left belt, while the right belt remains rigid. In this work, heart rate and energy expenditure are measured for healthy subjects in the challenging asymmetric environment created by the VST and compared to a traditional treadmill setting.

**Results:**

This study shows that this asymmetric environment results in an increase in heart rate and energy expenditure, an increase in activity in the muscles about the hip and knee, and a decrease in impact force at heel strike.

**Conclusions:**

Compliant environments, like those created on the VST, may be a beneficial tool as they can: reduce high-impact forces during running and walking, significantly engage the muscles surrounding the hip and knee allowing for targeted training and rehabilitation, and assist in identifying and training high fall-risk individuals.

## Introduction

The benefits of daily exercise have been thoroughly researched. Daily training of at least moderate intensity has positive correlations with improving sleep, mental health, self-confidence, memory, muscle strength, and bone strength, as well as negative correlations with stroke, stress, anxiety, obesity, and other cardiovascular and metabolic complications [1–5]. Running and jogging have become one of the most popular forms of cardio training with millions of people participating each year in the United States alone, either for leisure or competition [6–8]. Although daily exercise in the form of running is quite beneficial, there is always the risk of injury when performing physical activity. Studies have shown that the incidence of lower body injuries in runners can range anywhere from 19% to 73%. Many of these injuries occur at the knee, and it is thought that up to 50% of running injuries could be due to overuse [9–11].

It has been long believed that one of the main causes of overuse injuries in runners is high impact at initial contact [12]. Impact while running is defined as the high force between the ground and the foot, which can travel up the leg, affecting mainly the knees and hips [13,14]. Runners are often trained to reduce their impact force while running by altering form and mechanics throughout the gait cycle, mainly to reduce their risk of overuse injury [13]. In either avoidance of or recovery from overuse injuries, runners often opt for elliptical training sessions instead of running. While the elliptical has been shown to reduce vertical forces as compared to walking and running, it increases shear forces in the joints. Also, whether or not elliptical training is a suitable replacement for running training is still up for debate [15]. The difference in kinematics and kinetics may result in a reduction in training value as the movements may not translate well to running. Additionally, it is thought that these increases in shear forces and different ranges of motion could cause injury to those conditioned for running [15].

For jogging and running, there appears to be a need for a device that allows users to sufficiently raise their heart rate to train, while reducing impact force, and keeping similar kinematics to walking and running. This device would potentially be useful in training when preventing or returning from overuse injuries.

More generally, it appears that such a device would be valuable for those suffering from various joint injuries. After a joint injury occurs, the muscles surrounding the affected joint often become weak and need to be trained [16]. According to a 2021 study, it is estimated that 240 million people in the world have osteoarthritis at a level that limits their daily activity [17]. Osteoarthritis is most commonly seen at the knee and hip and has been linked to injuries earlier in life [18]. For the knee specifically, one school of thought currently believes that strong quadriceps may protect against developing and reducing the progression of osteoarthritis [19]. Another study supports this idea by stating that one of the best ways to rehabilitate the knee from common issues like “runner’s knee” and osteoarthritis is to strength train the hip [16]. For injuries directly affecting the hip joint, higher-intensity training is needed to strengthen muscles that have been weakened from the injury and inactive in early recovery [20]. With the number of individuals affected by hip and knee joint injuries, there appears to be a use for devices that require more work at the knee and hip joints, specifically the quadriceps. With most rehabilitation plans focused around isometric exercises to strengthen these muscles, there seems to be space for a device that is both functional in its task, such as walking or running, but still challenging enough at the hip and knee joints to promote muscle growth [19].

Additionally, a device comparable to the one described above could be useful for those who suffer from falls while walking. About 36 million falls happen each year, with 3 million resulting in trips to the emergency room [21]. Whether individuals fall because of neuromuscular disorder, physical inactivity, or old age, falls are extremely dangerous and limit one’s ability to move around safely and confidently. More recently, the idea of using challenging rehabilitation environments, in conjunction with a body-weight support harness, to identify and train those at risk of falling has been growing in popularity. One study has shown that environments that are challenging to walk in are great for identifying those at risk of falling. Subjects with a history of falling were difficult to identify in more standard smooth environments, but in environments with irregular or rough topography, fall-risk subjects were easily identified via various gait parameters [22]. Not only does this promote the idea of using difficult environments for identifying certain “at-risk” populations, but also suggests that being able to safely and easily walk in challenging environments could reduce falls in more standard environments. Likely, training subjects in these environments with the safety of a body-weight support harness could reduce the risk of injury in their daily activities. One group uses a challenging virtual reality environment with war veterans suffering from various gait issues. This environment has shown a lot of promise in its ability to push subjects and promote progress toward full or partial gait rehabilitation [23,24].

This study aims to propose the use of the authors’ unique robotic device, the Variable Stiffness Treadmill (VST), as a targeted gait training device. It is believed that this device has merit in all the areas described above: training runners while reducing joint impact force, intensely working the muscles surrounding the hip and knee joints in a rehabilitation context, and creating a challenging environment that would be valuable for identifying “fall risk” individuals and conditioning them for safe locomotion in their daily life. Previously, the VST has been investigated considerably as a robotic rehabilitation device [25–27], with one study even involving the use of virtual reality [28]. The results of this study show that the compliant environment created by the VST results in an increase in heart rate, overall energy expenditure, and work done at the hip and knee joints while reducing impact forces upon heel strike. The proposed system can revolutionize the ways robots can be used for targeted training for athletes or people suffering from an injury, as well as offer preventative training for those at risk of falling.

## Material and Methods

In this experiment, the Variable Stiffness Treadmill, detailed below, is tested as a targeted training device. Its merit as a training device will be explored by investigating energy expenditure, joint dynamics, muscle activity, and impact forces while walking in the unique environment the treadmill creates.

**Figure 1.**
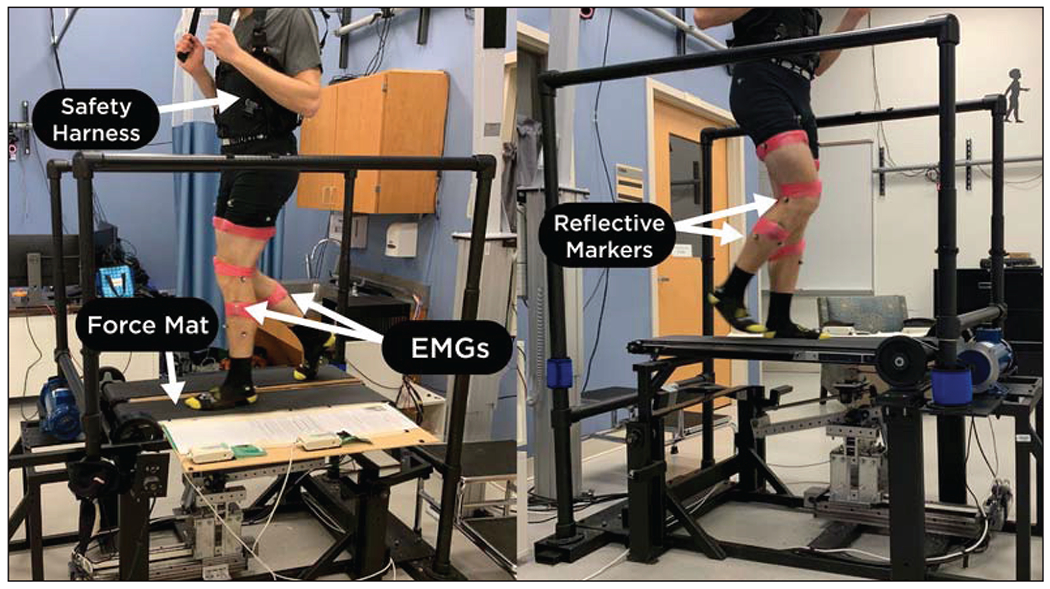
Subject walking on the Variable Stiffness Treadmill (VST). Reflective markers (used for motion capture), surface EMGs, the safety harness, and the force mat are labeled with arrows. The heart rate monitor cannot be seen since it is under both the harness and the subject’s shirt.

The Variable Stiffness Treadmill (VST) is the primary robotic device utilized in this study (Figure 1). The split-belt design of the VST allows the vertical stiffness of the left belt to be adjusted independently from the right belt. The approximate range of achievable stiffness values for the left belt is 60 N/m to 1 MN/m, with the latter value considered to be rigid. The right belt remains stationary and is unable to deflect vertically as the left belt can. As a result, various unilateral environments can be generated for subject locomotion where the right leg experiences a rigid stiffness while the left leg is exposed to a lower stiffness environment. Walking on the VST is comparable to walking with one foot supported on the pavement while the other steps on sand or similarly compliant terrains. Previous works further detail the capabilities and design of the VST [29,30].

### Experimental Protocol

Six healthy subjects free from musculoskeletal or neurological disorders impacting their walking or balancing abilities participated in this study (gender: 3 males, age: 24 ± 3.2 yrs, height: 1.7 ± 0.1 m, weight: 72.1 ± 5.2 kg). Informed consent was given by all participants before walking on the VST. The experimental protocol is approved by the Institutional Review Board at the University of Delaware (IRB ID# 1544521-2).

The two left belt stiffness values used in this experiment were 1 MN/m (rigid) and 30 kN/m while the right belt remained rigid throughout the experiment. As a comparison to the real world, 30 kN/m feels similar to walking on a soft yoga mat or sand. The stiffness of 30 kN/m was chosen after conducting pilot studies which indicated that this stiffness resulted in sufficiently different results as compared to the rigid condition without inducing major fatigue. The only two types of gait cycles were rigid (unperturbed) and those that involved the left belt having a constant stiffness of 30 kN/m during the left stance phase (perturbed). The left treadmill belt returned to a rigid state during the left swing phase to reduce oscillations following push-off. The subject is unaffected by this since they do not contact the left belt during the left swing phase. To ensure the left leg experiences the 30 kN/m stiffness during each perturbed gait cycle, the perturbation begins just before the left leg’s initial contact and ends just after the left leg’s lift-off.

The experiment began with a five-minute acclimation phase in which the subject walked on the treadmill with a mix of perturbed and unperturbed gait cycles. No data were used from this section as the purpose was solely to introduce the subject to the treadmill and the stiffness perturbations, as well as allow them to choose their desired speed. The subject had a choice of 90, 95, or 100 cm/s. They were asked to choose the speed that felt most comfortable and sustainable for over 20 minutes of walking.

The remaining duration of the experiment was split into two 23-minute sections. Each section began with a 90-second rest phase in which the subject relaxed in a chair to achieve a resting heart rate. The next 90 seconds involved the subject getting onto the treadmill and speeding up to their self-selected speed. No data were used from either of these sections, although the subject’s heart rate was monitored to ensure that the subject reached a resting heart rate level. During the remaining 20 minutes, subjects either walked with the left side of the treadmill set to rigid or with the left side set to 30 kN/m for each left stance phase. Data from this section of the experiment were analyzed. Half of the subjects walked with the unperturbed condition during the first 23-minute trial before walking in the perturbed condition during the second 23-minute trial. The other half walked in the perturbed condition first, followed by the unperturbed trial. This was to ensure that fatigue during the second trial was not a factor during data analysis.

Each subject had 22 passive motion capture markers adhered to their lower body to track their leg motion. Eight VICON cameras recorded the location of the markers with a frequency of 100 Hz. The activity of five major muscles in each leg was recorded using ten wireless surface electromyographic (EMG) electrodes (Trigno, Delsys Inc.) following ISEK guidelines [31]. The contact material of the electrodes used is 99.9% Ag. Also, the electrodes are rectangular in shape, the contact dimensions are 5mm-by-1mm, and the inter-electrode spacing is 10mm. Prior to application, the skin was shaved, if necessary, and cleaned with an alcohol wipe. The EMG data was recorded at a frequency of 2 kHz. The recorded muscles were the tibialis anterior (TA), gastrocnemius (GA), vastus medialis (VA), rectus femoris (RF), and biceps femoris (BF). A heart rate monitor (Polar H10) recorded the subject’s heart rate at a 1 Hz frequency. The motion capture and EMG data were synchronized via a trigger signal. To synchronize the heart rate data, the trial was simply started at the same time as the motion capture data. With a sampling rate of 1 Hz, this was sufficiently accurate. A body weight support harness was worn by all subjects as a safety precaution when walking on the treadmill, but it did not offset any weight. Additionally, the VST is equipped with force mats (Tekscan 3,510 Medical Sensors) under the left belt of the treadmill to track force distribution as the subject walks. Unfortunately, the force mats were not functional at the time of this data collection. Force mat data will be supplemented from a very similar study. The details of this will be discussed in the “Impact Force from Supplemental Study” subsection below.

Subjects were asked to keep their arms above their lower limbs during the experiment to ensure the consistent visibility of all motion capture markers. This was achieved by either keeping their elbows flexed while swinging their arms or resting the backs of their hands on the handrails. The latter option discouraged subjects from offsetting weight onto the handrails or using it as a balancing tool. Subjects were also informed that they could grasp the handrails or request to stop the experiment if they ever felt unsafe, but neither event ever occurred.

### Data Processing

After the raw kinematic, muscular activity, and heart rate data were synchronized, heel strikes were detected using the F-VESPA algorithm [32,33]. The time from the left heel strike to the following left heel strike was defined as a single gait cycle. Outlier gait cycles were detected using a systematic method, which examines kinematics and muscle activity data [34]. An average of 22.8 ± 7.0 outlier gait cycles were identified per trial per subject. The stance and swing phases of both the left and right legs were established using the detected heel-strike events and toe-off events, which were calculated by finding when the toe marker was most posterior.

Statistical analysis was performed using the Wilcoxon rank sum test (the non-parametric version of the t-test) [35]. This test was chosen as it does not assume any distribution of the data, such as a normal distribution or beta distribution. An α value of 0.05 was used for all significance tests. Note that statistical significance is denoted by bold text and statistical tests are only able to be performed for the heart rate analysis as all energy calculations result in a single value, which is not able to be significance tested. Percent increase will be presented in all tables though.

A 4th-order Butterworth band-pass filter was used to process EMG data using a low cut-off frequency of 30 Hz and a high cut-off frequency of 300 Hz. After full-wave rectifying the data, the signal envelope was established with a moving average window of 400 data points (200 ms). A 4th-order Butterworth low-pass filter was used to filter the data one final time with a cut-off frequency of 5 Hz. Following this processing, the maximum EMG data value from each muscle across both the perturbed and unperturbed trials was used to normalize the EMG data. Finally, heart rate data were filtered with a 2nd-degree polynomial local regression with a window of 50 data points.

### Energy Estimate Calculations

Due to energy expenditure being a main focus of this study, it was estimated using three different data types: heart rate, dynamics, and muscle activity data. While each strategy is certainly related, resulting in some level of redundancy, each strategy also has unique limitations. While the purpose of this work is not to compare these methods, testing and presenting all three strategies allows for a more thorough analysis. First, expended energy *E*_*HR*_ was estimated via heart rate using approximated functions found in the literature (36):

(1) $$E_{\mathrm{HR}}=\{\begin{array}{cc} \int_{0}^{t_{f}}(a+bH+cW+dA)dt, & if\;male\\ \int_{0}^{t_{f}}\left(f+gH+hW+iA\right)dt, & if\;female \end{array}$$

where *t_f_* is 20 minutes (i.e., the length of the trial), *H* is the instantaneous heart rate, *W* is the subject’s mass, *A* is the subject’s age, while the parameters α=−55.0969, *b*=0.6309, *c*=0.1988, *d*=0.2017, −20.4022, *g*=0.4472, *h*=−0.1263, and *i*=0.074 are constants taken from the literature [36]. Expended energy over the entire trial can be determined by simply taking the integral with respect to time as shown in (Equation 1).

Second, energy expenditure was estimated via lower body dynamics. To accomplish this, each leg was treated as a three-segment rigid body with the hip, knee, and ankle able to move with 3 degrees of freedom. Using the subject’s height, weight, and leg dimensions, the Nexus software package (VICON) estimates the inertia of each segment, and the total energy expenditure of the lower body can be calculated as:

(2) $$E_{DYN}=\sum_{j=1}^{6}\int_{0}^{t_{f}}\left(|I_{j}\alpha_{j}\omega_{j}|\right)dt$$

where $E_{DYN}$ is total expended energy using this method, *j* represents each of the 6 major joints of the lower body, *t_f_* is 20 minutes, *I* is the moment of inertia of the related links, α is the angular acceleration, and ω is the angular velocity of these joints.

Last, a value proportional to energy expenditure was approximated using EMG data:

(3) $$E_{EMG}=\sum_{m=1}^{10}\int_{0}^{t_{f}}\left(|x_{m}^{2}|\right)dt$$

where *E_EMG_* is a value proportional to metabolic energy, *m* represents each of the 10 muscles for which muscle activity data was collected, *t_f_* is 20 minutes, and is the filtered muscle activity data. Additional parameters about each subject’s muscles, such as maximum force, would need to be collected to give an actual energy estimate, which was not possible for this experiment. However, since the goal is to compare the energy between two conditions, describing the energy expenditure as proportional to the EMG activity of the most relevant muscles should allow for an accurate comparison between the two conditions.

## Results

This study shows that the unilaterally compliant environment created on the Variable Stiffness Treadmill (VST) requires more energy to walk on than a typical rigid treadmill. Additionally, this environment targets certain muscles and muscle groups causing an increase in activation. Last, the compliant environment seems to lead to a reduction in impact at heel strike. These results will be examined in depth below and further considered in the Discussion section. Note that in all graphs, red will represent the rigid phase of the experiment, while blue will represent the compliant phase. Subject information and metrics are displayed below in Table 1 to give more context throughout the results.

### Total Energy Expenditure

**Figure 2.**
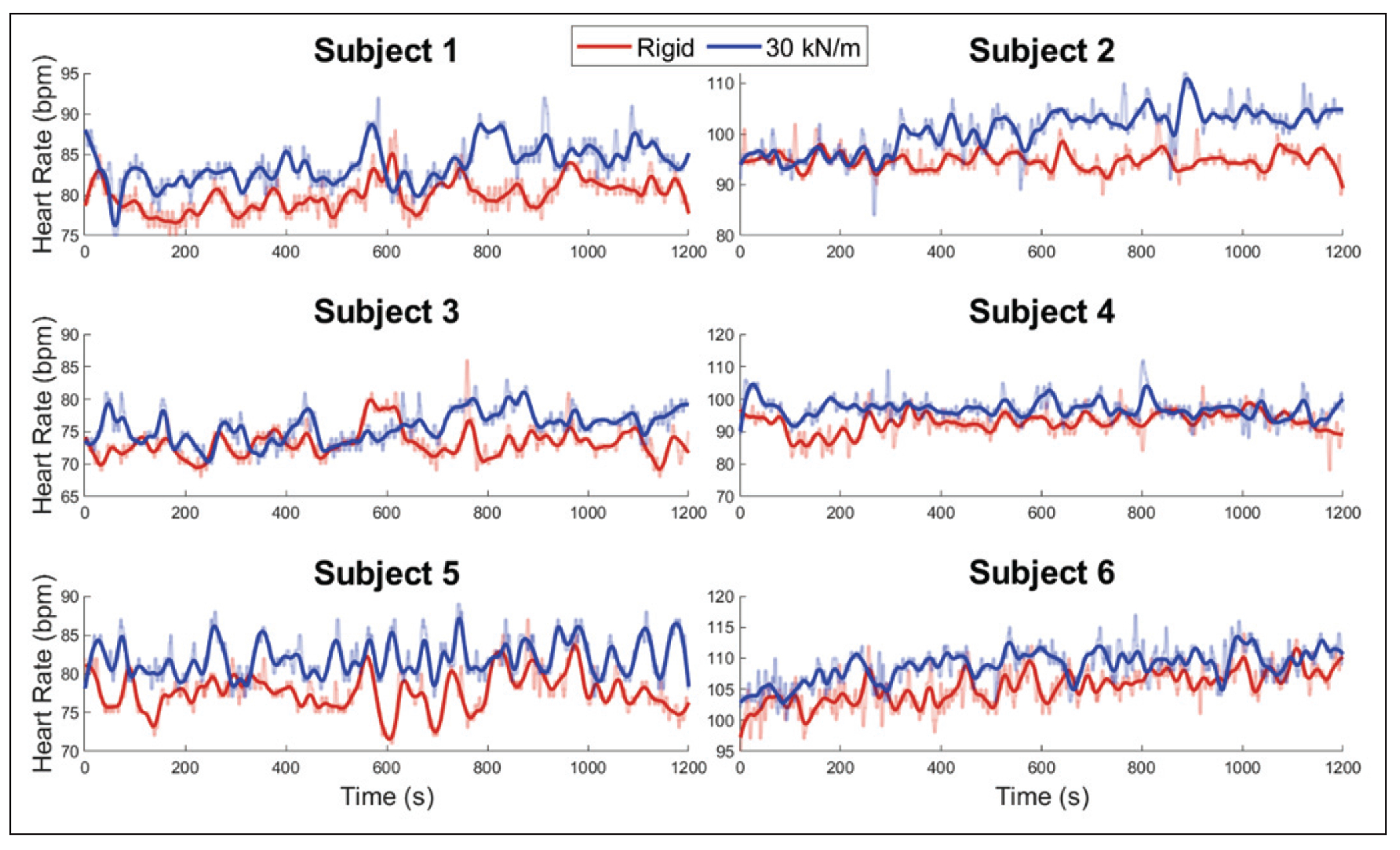
Heart rate for each subject across the entire 20-minute experiment. Data were filtered via 2nd-degree polynomial local regression with a window size of 50 data points. Unfiltered data can be seen by the lighter lines.

First, the unilaterally compliant terrain tested in this study resulted in an increase in heart rate for all six subjects. This can be seen graphically in Figure 2 and numerically in Table 2. Note that a statistically significant increase (a p-value of less than 0.05) is denoted using bold font. A percent increase of 3.5% to 6.2% from rigid to unilaterally compliant terrain was present for all subjects with an average of 4.6%. Increases from rigid to compliant were statistically significant for all subjects.

Next, using heart rate, energy expenditure was calculated in Table 3. An even greater increase was found with respect to energy as compared to heart rate, with percent increase values ranging from 7.38% to 19.69%. An average percent increase of 15.27% was seen across all six subjects.

Energy was also estimated via dynamics at each major joint on the lower body (left hip, right hip, left knee, right knee, left ankle, right ankle) and summed together to estimate total energy. These joints will be examined individually in the section below. For five out of six subjects, energy expenditure increased, while a slight decrease was seen in one subject. The percent increase ranged from −2.03% to 30.17% with an average of 16.13%, which is comparable to the energy estimation based on heart rate. It must be noted that a direct comparison of this energy estimate with the heart-rate-based estimate is not important since only lower-body dynamics were used in this method. The method for comparison between the two conditions (Rigid and Compliant) yielded similar observations.

### Energy Expenditure by Joint & Muscle

These increases in energy expenditure can be understood on a deeper level by looking more closely at individual joints and muscles. With respect to dynamics, individual joints were investigated. With respect to muscle activity data, all 10 observed muscles were investigated both numerically and via their profiles throughout the gait cycle.

First, using the body dynamics as described above, energy expenditure was estimated at each of the major joints in the lower body. These results can be seen in Table 4. Recall that the overall increase in energy expenditure using the dynamic estimation method was on average 16.13%. As can be seen, most joints are slightly below this average value, those being the right hip, right knee, left ankle, and right ankle. The left knee is quite close to the average value, while the left hip has a 41.84% increase. According to these estimates, the left hip is responsible for over 50% of the extra energy expended in the lower body. This will be investigated in further detail below with muscle activity data.

Next, an increase in energy expenditure can be estimated for each muscle using (Equation 3). This data can be seen in Table 5. The “Rigid” and “Compliant” columns do not have units as these are only values proportional to the metabolic energy expended by each muscle. All muscles except the right GA and the right BF show an increase in energy expended. All other muscles, except for the left TA, show a large increase of at least 10%. The left RF, right RF, and left GA show the largest increase of 63.41%, 45.26%, and 36.21%, respectively.

**Figure 3.**
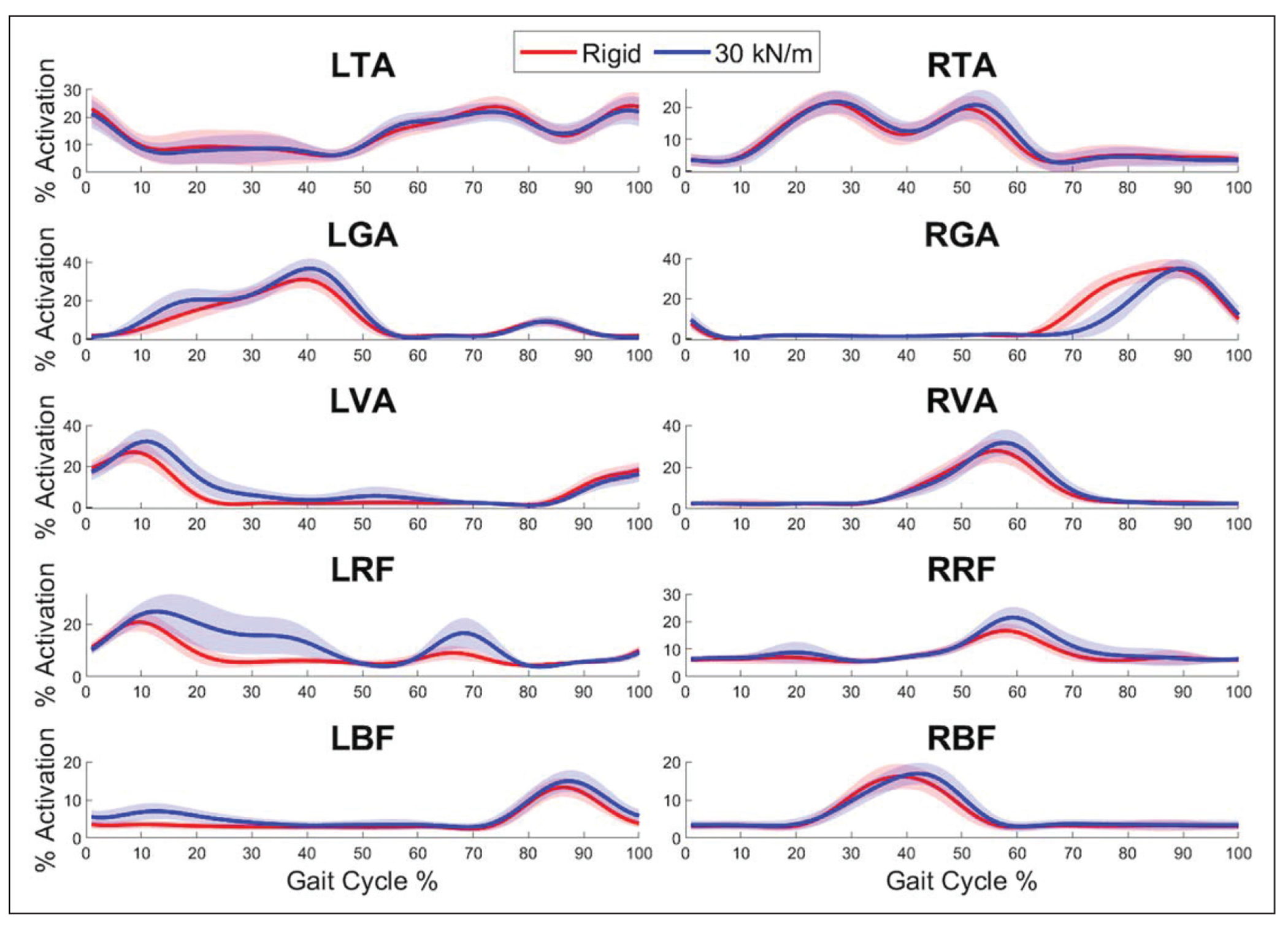
Muscle activity data from a representative subject averaged with respect to a gait cycle. Solid lines show average data per gait cycle, while shaded areas are one standard deviation above and below the average.

Lastly, each muscle can be evaluated over the gait cycle (Figure 3). The data shown are from a representative subject. The left TA shows very little change when going from a rigid terrain to a unilaterally compliant terrain. With respect to gait cycle percentage, the right TA shows an increase from about 30% to 40% and from about 50% to 65%. Respectively, these sections are the terminal swing phase and loading response for the right leg. Next, the left GA shows an increase for nearly the entire first half of the gait cycle. This represents the time from initial contact to terminal stance, which is nearly the whole left stance phase. The right GA, however, shows the opposite trend of decreased activation during the right stance phase. The left VA shows increased activation for the entire left mid-stance sections of the gait cycle (10% to 30%). The right VA shows a similar trend of increasing during the right mid stance. The left RF shows a substantial increase during the right stance and right initial swing. Next, the right RF activity is increased during the loading response of the right leg. The left BF shows increased activation from terminal swing through loading response. Last, the right BF shows slightly delayed activation during the right terminal swing, but not a notable increase or decrease in activation level.

### Impact Force from Supplemental Study

While impact force was not able to be tested during this study, it was able to be studied during a similar experiment. The data presented below is supplementary and should be viewed as such [26].

**Figure 4.**
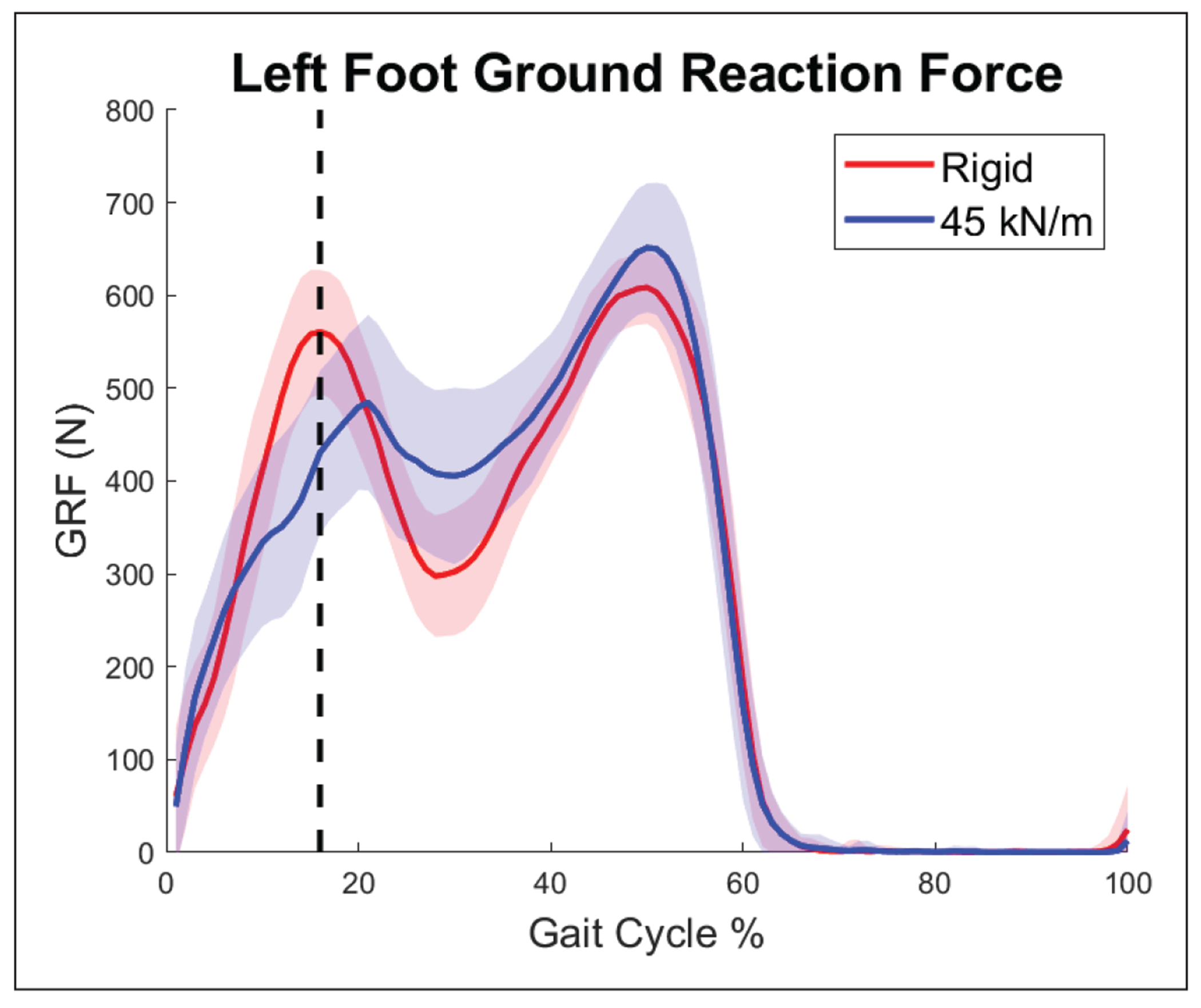
Averaged ground reaction force of a single subject during a gait cycle starting at left heel strike. The vertical dashed line denotes the end of the rising edge of the force profile for the rigid case. Solid lines show average data per gait cycle, while shaded areas are one standard deviation above and below the average.

Impact force upon heel strike was investigated through another experiment which had a very similar layout. In this study, subjects first walked on the VST with both sides set to rigid for about 5 minutes. Then, subjects walked with the left side of the treadmill set to 45 kN/m for approximately 10 minutes. Due to the very similar experimental layout, identical equipment usage, and being performed by the same researcher, the data from this supplementary experiment can be insightful in understanding the compliant terrain discussed in this study [26]. The force mats captured force data from 2068 sensors at a frequency of 60 Hz. The data was synchronized with motion capture data using a trigger signal. Data from a representative subject shows that impact force during and right after initial contact is reduced (Figure 4). The vertical dashed line shows where the rising edge of the force in the rigid profile ends. The compliant scenario not only has a reduced peak value but also spreads the force over a greater amount of time.

## Discussion

The results of this study suggest that compliant environments may be beneficial for preventing overuse injuries, re-habbing existing injuries, and avoiding injuries through fall detection and prevention. In this section, these applications are further discussed, as well as the shortcomings of this work and plans for future studies.

### Gait Training Applications

First, training in compliant environments may have advantages over training on rigid ground. The increase in heart rate for all six subjects most directly demonstrates the value of using compliant environments for training. This environment, at least in the unilateral format seen in this study, is more stressful on the heart and can be safely assumed to require more energy to walk in. This estimate is further confirmed in five of six subjects using dynamics to estimate energy usage. It should be noted that subject six does appear to be an outlier but was not removed from this study. It is hypothesized for this subject that either the 90-second rest period between trials was not sufficient, or the experiment was too challenging and pushed the subject into a different heart rate zone, resulting in a different response. For ground reaction force, a decrease is mainly seen during initial contact. This is the highest impact portion of the gait cycle, and minimizing initial force is thought to be valuable in preventing injury during walking or running [12,13]. The combination of increased metabolic activity and decreased impact suggests training in compliant environments could be more efficient and safer.

Second, this unilaterally compliant environment, along with the benefits discussed above, targets certain muscles. Large increases in muscle activity data can be seen in the left VA and RF when comparing compliant data to rigid. They both increase during the first half of the gait cycle, which is during the left stance phase. Since both muscles are knee extensors, they both appear to be activating more to keep the knee from buckling in this unfamiliar and unstable environment. The left RF is also activating more during the left swing phase, which is most likely contributing toward an increased leg swing speed as it is a hip flexor. Large increases are also seen in the left GA and BF during the left stance phase, which could be for a similar reason as the VA and RF. Both the GA and BF are knee flexors, possibly working to keep the knee from hyperextension. Similarly, on the right side, the increases in VA and RF activity could also be working toward speeding up the right swing phase. This possibly occurs to minimize the time spent in left single support, in the unfamiliar compliant environment. Regardless of the mechanisms causing these increases, this targeted training to the muscles at the hip and knee joints could be a beneficial form of rehabilitation or reentry into training after an injury [16].

Note that the results of increased energy expenditure and increased muscle activity do agree with studies in adjacent areas. While compliant terrains, like that studied in this work, are largely unexplored, gait across other challenging terrains has been well documented. Surfaces classified as slippery, rocky, and uneven, have been shown to increase knee and hip flexion and increase overall gait variability, most likely pointing toward a lower level of energy efficiency [37,38]. These terrains have been shown to be harder for older individuals but still appear to be difficult for people of all ages.

Third, it is hypothesized that this compliant environment, either unilateral or bilateral, could be valuable in predicting and training fall-risk individuals. This was not directly investigated in this study, as it will be in future studies, but this did show that compliant environments can be more taxing with respect to energy and muscle activity. It has been shown that challenging environments, such as uneven surfaces were effective in identifying fall-risk subjects [22]. It is thought that the challenging environment studied in this work can create safe [body-weight-supported] and low-impact scenarios that could be valuable in predicting and preventing falls [23,24].

### Shortcomings

There were many shortcomings in this study that should be noted. First, it is possible that training in these asymmetric and/or challenging environments could result in compensatory mechanisms or disturb coordination. This would obviously need to be investigated further in long-term studies, but it is hypothesized at this point to not be an issue, as sand is currently known to be a safe and beneficial terrain for training [39]. Second, only six subjects are tested in two different environments, rigid and compliant (30 kN/m). For a well-rounded, fuller understanding of using low-stiffness environments for training, more subjects in a variety of conditions would need to be examined. It is plausible that different levels of compliance could have a different magnitude of effect on subjects, or even a totally different type of effect. Additionally, all six subjects in this study were young and healthy, and since many of the proposed use cases pertain to older or injured subjects, they should be evaluated as well. Last, since impact force could only be explored tangentially by using another study, it should be more directly investigated as well.

### Future Studies

Moving forward, multiple studies will be conducted with different subject populations in both unilaterally and bilaterally compliant environments. These studies will also include a wider age range in the subject pool, as well as both healthy and injured subjects. As stated above, impact force needs to be studied more directly, and will be collected in future studies for both left and right legs. Future studies will also begin to investigate different modes of locomotion such as speed walking, jogging, and running. These studies will further explore the three use cases of preventing overuse injuries, targeted rehabilitation, and fall-risk detection and prevention.

## Conclusions

This paper suggests that walking and running in compliant environments could be useful in a variety of scenarios. First, the reduced ground stiffness creates an environment for runners to train without fear of overuse injuries. This also allows runners to ease back into training after overuse injuries, all while generating a cardio workout more intense than standard treadmill training. Second, an asymmetrically compliant environment, like that created on the Variable Stiffness Treadmill (VST), requires more work from the quadriceps, hamstrings, and other muscles around the hip. This is a promising form of rehabilitation for those who suffer from injuries such as osteoarthritis or “runner’s knee,” since it allows for targeted training of specific muscle groups. Third, this rather challenging walking environment may be useful for identifying and training those who are at risk of falling. Difficult walking environments have been shown to be better for identifying “fall-risk” individuals. Having these individuals walk in these environments with body-weight support, like that seen on the VST, would allow individuals to train in this unique environment without the risk of falling. All these use cases are accompanied by the health benefits of an increased heart rate, and the common functionality of walking.

## Figures and Tables

**Table 1.** Subject Information and Metrics

	Sex	Age (years)	Height (m)	Weight (kg)
**Subject 1**	Female	26	1.57	63.2
**Subject 2**	Female	20	1.70	77.1
**Subject 3**	Male	27	1.78	73.7
**Subject 4**	Female	26	1.57	68.8
**Subject 5**	Male	25	1.80	74.5
**Subject 6**	Male	20	1.73	75.3

**Table 2.** Heart Rate Data Comparison Between Rigid and Compliant Conditions

	Rigid (bpm)	Compliant (bpm)	% Increase
**Subject 1**	79.9 +/− 2.0	**83.8 +/− 2.6**	4.8
**Subject 2**	94.9 +/− 2.1	**100.8 +/− 4.4**	6.2
**Subject 3**	73.3 +/− 2.5	**75.8 +/− 2.5**	3.4
**Subject 4**	92.9 +/− 3.6	**97.1 +/− 3.3**	4.5
**Subject 5**	77.7 +/− 2.6	**82.0 +/− 2.4**	5.5
**Subject 6**	105.3 +/− 3.0	**108.9 +/− 2.8**	3.4

**Table 3.** Energy Expenditure Estimated via Heart Rate

	Rigid (kJ)	Compliant (kJ)	% Increase
**Subject 1**	185.70	219.75	18.34
**Subject 2**	275.57	328.18	19.09
**Subject 3**	224.31	255.91	14.09
**Subject 4**	287.45	324.88	13.02
**Subject 5**	275.73	330.02	19.69
**Subject 6**	606.61	651.39	7.38

**Table 4.** Energy Expenditure Estimated via Dynamics per Subject and per Joint

	Rigid (kJ)	Compliant (kJ)	% Increase
**Subject 1**	7.00	8.86	26.59
**Subject 2**	7.53	9.81	30.17
**Subject 3**	6.92	8.51	23.03
**Subject 4**	6.08	6.57	7.99
**Subject 5**	8.04	8.93	11.04
**Subject 6**	6.75	6.62	−2.03
			
**Left Hip**	1.79	2.54	41.84
**Right Hip**	1.92	2.11	9.89
**Left Knee**	1.52	1.79	18.03
**Right Knee**	1.47	1.63	10.47
**Left Ankle**	0.21	0.24	10.67
**Right Ankle**	0.20	0.23	14.70

**Table 5.** Energy Expenditure Estimated via EMG Data by Muscle

	Rigid (*10^7^)	Compliant (*10^7^)	% Increase
**Left TA**	2.29	2.31	0.91
**Right TA**	1.09	1.25	14.91
**Left GA**	1.20	1.63	36.21
**Right GA**	1.78	1.32	−26.25
**Left VA**	0.73	0.82	11.62
**Right VA**	0.84	1.07	28.06
**Left RF**	0.61	1.00	63.41
**Right RF**	0.65	0.95	45.26
**Left BF**	0.20	0.25	24.69
**Right BF**	0.44	0.43	−3.11
